# Cytomegalovirus and Pregnancy: A Narrative Review

**DOI:** 10.3390/jcm13020640

**Published:** 2024-01-22

**Authors:** Karina Felippe Monezi Pontes, Luciano Marcondes Machado Nardozza, Alberto Borges Peixoto, Heron Werner, Gabriele Tonni, Roberta Granese, Edward Araujo Júnior

**Affiliations:** 1 Department of Obstetrics, Paulista School of Medicine, Federal University of São Paulo (EPM-UNIFESP), São Paulo 04023-900, SP, Brazil; karinamonezi@hotmail.com (K.F.M.P.); lunardozza@uol.com.br (L.M.M.N.); araujojred@terra.com.br (E.A.J.); 2 Service of Gynecology and Obstetrics, Ipiranga Hospital, São Paulo 04262-000, SP, Brazil; 3 Gynecology and Obstetrics Service, Mário Palmério University Hospital, University of Uberaba (UNIUBE), Uberaba 38050-501, MG, Brazil; albertobpeixoto@gmail.com; 4 Department of Obstetrics and Gynecology, Federal University of Triângulo Mineiro (UFTM), Uberaba 38025-180, MG, Brazil; 5 Department of Fetal Medicine, Biodesign Laboratory DASA/PUC, Rio de Janeiro 21941-901, SP, Brazil; heron.werner@gmail.com; 6 Department of Obstetrics and Neonatology, Istituto di Ricovero e Cura a Carattere Scientifico (IRCCS), AUSL Reggio Emilia, 242100 Reggio Emilia, Italy; 7 Obstetrics and Gynecology Unit, Department of Human Pathology of Adult and Childhood “G. Barresi”, University Hospital “G. Martino”, 98124 Messina, Italy; rgranese@unime.it; 8 Discipline of Woman Health, Municipal University of São Caetano do Sul (USCS), Campus Center, São Caetano do Sul 09521-160, SP, Brazil

**Keywords:** cytomegalovirus, pregnancy, transmission, serology, treatment

## Abstract

Cytomegalovirus (CMV) infection is the most common congenital infection worldwide, affecting between 0.7% and 1% of all live births. Approximately 11% of infected newborns are symptomatic at birth, and between 30% and 40% of these are at risk of developing long-term neurological sequelae. Until recently, the lack of an effective treatment did not justify universal testing of pregnant women. In recent years, however, valacyclovir at a dose of 8 g/day has been shown to be effective in preventing vertical transmission, and ganciclovir has been shown to be effective in preventing long-term sequelae in the treatment of symptomatic neonates. The aim of this article is to review congenital CMV infection, from its epidemiology to its treatment, using the most recent studies in the literature, and to help in the decision to modify protocols for universal testing of pregnant women according to the possibilities of each locality.

## 1. Introduction

Cytomegalovirus (CMV) is an enveloped DNA virus that, like other members of the herpes virus family, establishes a lifelong latency period after primary infection and becomes resident in monocytes and granulocytes [1,2]. For this reason, vertical transmission can occur through primary infection, reactivation of the disease, or even contamination with another strain [2]. CMV infection is spread through contact with contaminated bodily secretions (such as urine, saliva, genital secretions, and breast milk) and generally causes few symptoms in immunocompetent individuals, but can cause serious damage in immunosuppressed individuals, including fetuses [2,3].

CMV is the most common congenital viral infection in the world [1,2,3,4,5,6,7,8,9,10,11,12,13,14,15,16,17,18,19,20,21,22,23,24,25,26,27,28,29,30,31,32,33,34,35,36,37,38,39,40,41,42,43,44,45,46,47,48,49,50,51,52,53,54,55,56,57,58,59,60,61,62], with a prevalence rate among all live births of approximately 0.5% to 2% [1,2,3]. It is the leading cause of permanent sequelae, responsible for 25% of cases of congenital sensorineural hearing loss; 10% of cases of cerebral palsy; and severe neurological abnormalities, vision loss, and growth retardation [1,2,3]. In the United States, approximately 8000 children per year are diagnosed with neurological sequelae of congenital CMV infection, only half of which are related to primary maternal infection [22]. This figure is higher than many other well-known childhood and genetic diseases combined [23]. The estimated annual cost of sequelae of congenital CMV infection in the United States is approximately $2 billion [29].

In a recent meta-analysis, Zuhair et al. [4] estimated the global prevalence of CMV seroprevalence to be 83% in the general population and 86% in women of childbearing age, with the latter reaching 90% in Brazil. According to Swanson et al. [2], despite the high prevalence and serious consequences of congenital CMV infection, the disease is poorly understood by the general population compared to other, rarer conditions such as Down’s syndrome, fetal alcohol syndrome, and spina bifida. This fact shows that health professionals and governments do not carry out prevention and public awareness campaigns, which would be the only way to prevent this comorbidity, since there is no vaccine available (although several clinical trials are underway), and the available treatment is not yet universally accessible or accepted by the scientific community as a whole [2,4].

Promising studies on the treatment of pregnant women with seroconversion in the periconceptional period and in the first trimester of pregnancy with high doses of valacyclovir (8 g/day) have changed the guidelines for active research on the disease in prenatal care via serology (until recently not indicated in any country) and treatment [3].

The purpose of this article is to review congenital CMV infection, from its epidemiology to the available treatments, to help health care professionals and health authorities make decisions about whether to routinely implement CMV serologic testing for pregnant women and whether to initiate treatment during pregnancy.

## 2. Epidemiology

CMV is a DNA virus of the herpes virus family, and it has no seasonality [2]. The global prevalence of CMV is 83% (95% CI, 78–88) in the general population, 86% in women of childbearing age (95% CI, 83–89), and 86% in blood or organ donors (95% CI, 82–89) [5]. The study also showed that the prevalence is higher in lower socioeconomic groups [56]. These data are important because the estimated seroconversion in pregnant women is higher than the prevalence of CMV in the general population [1,2,3,4,5]. It is known that CMV infection caused by primary infection has a greater capacity for vertical transmission and greater potential for severe congenital infection [1,2,3,4,5].

After primary infection, the virus can remain latent for years and reactivate (non-primary infection/reactivation), or the same individual may be infected with a different strain (non-primary infection/reinfection) [1,2,56,62]. The majority of infected newborns worldwide are born to previously infected women (non-primary infection); in other words, a community with a high prevalence of CMV contributes to an increased risk of all three forms of infection [4,56].

Seroprevalence in developing countries is about 90% in adolescents and 95% in young adults, which explains the higher rate of congenital CMV infection in pregnant women with non-primary infection [5,56,62]. In comparison, seroprevalence in the United States is 40–60% in 12–40-year-olds [5]. The rate of reinfection with a new strain in previously exposed women is about 18–30%, suggesting that reinfection could be a major cause of non-primary infection [6]. Mussi-Pinhata et al. [7] published a study in Brazil to determine the risk of seroconversion in pregnant women in a population with a high prevalence of CMV seropositivity. The cumulative rate of seroconversion (among previously nonimmune patients) was 13.9% (95% CI, 4.8–30.6). Congenital CMV infection was 2.8% in the newborns of pregnant women with primary infection and 0.5% in those who were seropositive prior to pregnancy [7]. In this study, consistent with worldwide statistics, the majority of affected newborns were born to mothers with pre-existing immunity, although the risk was higher for primary infection (1 of 36 vs. 8 of 1685 newborns) [7].

The greatest risk factor for CMV transmission in women of reproductive age is exposure to the contaminated saliva and urine of young children, with a risk up to 10 times higher than in other unexposed groups [2,42,50,52].

Congenital CMV infection is the most common congenital infection and the major cause of sensorineural hearing loss in early childhood and mental retardation in the absence of genetic alterations [1,2,3,4,5,6,7,8,9,10,11,12,13,14,15,16,17,18,19,20,21,22,23,24,25,26,27,28,29,30,31,32,33,34,35]. Its incidence varies from 0.5 to 2.0% in newborns, with wide variability between countries or even between hospitals in the same region [1,2,3,4,5,6,7,8,9,10,11,12,13,14]. For example, in a study conducted in Gambia, the incidence of congenital CMV was 13.6% compared to 0.46% in Sweden [1,2]. The prevalence of infected newborns among all CMV-positive women is approximately 1%, although the rate may be as high as 3.4% among reinfected women. Seronegative women living in areas with low CMV seroprevalence have an infection rate of 1–3%, but placental transmission in these cases is 30–50% [6].

The prevalence of live births with congenital CMV is three times higher in countries of low to medium socioeconomic status than in rich countries [47]. A recent study estimated the direct costs (hospitalization, medication, inpatient visits, etc.) and indirect costs (social costs, care, school and family support, etc.) of congenital CMV. In Japan in 2019, the total cost was 27.6 billion yen (4% direct cost); in the United Kingdom in 2016, 324 million pounds sterling (40% direct cost); and in Germany, 70.5 million euros/year (8% direct cost) [39].

## 3. Maternal Infection

### 3.1. Contamination

CMV contamination occurs through direct contact of the mucous membranes with contaminated body fluids, such as urine, saliva, blood, genital secretions, tears, contaminated breast milk, solid organ transplants, and stem cells [8,9,30]. The major risk factor for maternal infection is contact with children younger than 2 years, who can shed the virus in saliva and urine for up to 24 months [1,8,50]. Another significant route is sexual transmission [1]. There are 3 types of infection: primary, when the mother has previously tested negative for CMV (IgG and IgM) and seroconversion occurs during pregnancy; reactivation of latent virus; and contamination with a new strain in patients with previous contact, the last two of which are considered non-primary [1,2,3,4]. All 3 types of infection can lead to vertical transmission [1,2,3,4,8,30,59].

An interesting finding from a systematic review of the literature is that although the rate of CMV infection is higher among childcare workers, the rate is not high among healthcare workers. This finding may suggest that extra care is not needed for pregnant women who belong to the latter category [42].

### 3.2. Symptomatology

CMV infection generally causes minimal or no symptoms in immunocompetent individuals, but can cause serious illness in immunosuppressed individuals (HIV-positive, transplant patients, immunosuppressant users, and fetuses) [1,9]. In immunosuppressed individuals, viral replication tends to be uncontrolled, which is associated with viremia and dissemination to several organs and can lead to pneumonitis, hepatitis, retinitis, or gastroenteritis [4,9].

### 3.3. Screening

In 2023, a systematic review of the literature carried out by Xie et al. [8] on the existence of guidelines and consensuses for CMV screening during pregnancy found that as of June 2022, none of the 13 included studies suggested universal screening. Eight guidelines and 2 consensuses were against universal testing in this population. The UK’s Royal College of Obstetricians and Gynaecologists recommends universal screening for research purposes only, while the Society of Obstetricians and Gynaecologists of Canada accepts universal screening if the IgG avidity test is available. Five guidelines recommend targeted screening only for patients at high risk of infection, i.e., pregnant women who have children up to 3 years of age or who work in daycare centers [8]. However, the guidelines differ on how this testing should be performed, noting 2 types of approaches: the first using IgG, IgM, and IgG avidity testing, and the second using only specific IgG testing. The study by Xie et al. [8] was limited to English language guidelines, and 10 others were excluded due to translation difficulties.

According to Fowler et al. [9] in a systematic review of the literature published in 2022, the rate of seroprevalence of IgG immunoglobulin for CMV in women of reproductive age varies between countries and continents, with 45.6–95.7% in Europe, 60.2% in Japan, 58.3–94.5% in Latin America, and 24.6–81% in North America. Seroprevalence increases with age and is higher in developing countries than in developed countries. The same study found a heterogeneous prevalence of IgM immunoglobulin for CMV in women of reproductive age: Europe, 1–4.6%; North America, 2.3–4.5%; Japan, 0.8%; and Latin America, 0–0.7% [9].

### 3.4. Serologies and Interpretations

**Figure 1:**
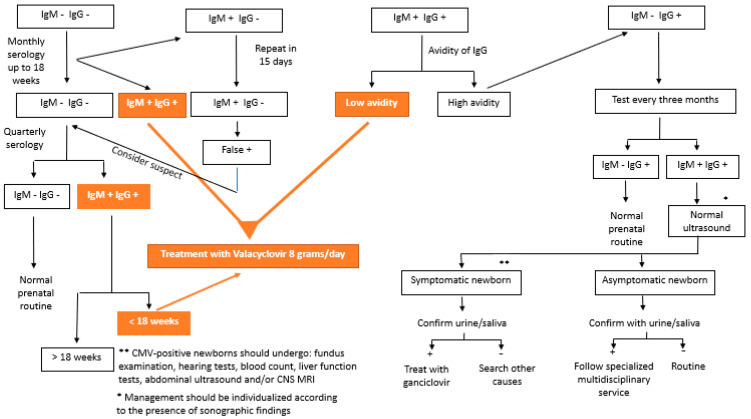
Flowchart of the serology screening of intrauterine cytomegalovirus infection until 14 weeks of gestation.

CMV testing can be performed by testing for specific antibodies (IgG, IgM, and IgG avidity) or by detecting cytomegalovirus DNA in body fluids (blood, urine, and saliva) [3,56]. In 2020, Maltezou et al. [10] suggested interpreting combinations of the results of these serologies in the case of fetal infection, as shown in Table 1. Figure 1 shows the flowchart of serology screening of intrauterine CMV infection until 14 weeks of gestation.

## 4. Congenital Infection

### 4.1. Transmission

The average worldwide prevalence of newborns with congenital CMV infection is 0.64% and can reach 1% in some populations [1,6]. The prevalence of newborns affected by CMV is lower among those whose mothers were serologically tested during prenatal care (0.48%) than among newborns tested only at birth (0.70%). This may be due to greater care to avoid contamination and/or termination of pregnancy if fetal contamination is suspected [1].

Primary CMV infection during pregnancy appears to be the greatest risk factor for congenital infection, with approximately 30–40% of these fetuses being infected at birth [1,2,6,17]. In contrast, only 1% to 3% of newborns from mothers with non-primary infection are affected [1,2,6]. CMV infection can also occur intrapartum or postnatally through exposure to cervical secretions during childbirth or through breastfeeding, but these types of infection rarely cause symptoms or sequelae in term newborns [2]. Rates of transmission to the fetus in primary maternal infection also vary with the trimester of maternal infection, ranging from 20–30% in the first trimester to 72% in the third trimester of pregnancy [6,10,11].

Breast milk infected with CMV, when given to preterm and low-birthweight newborns, appears to have an increased rate of transmission and can cause symptoms. To reduce the risk, the milk can be treated (pasteurized), but this is not done in full-term and term infants, and further studies are needed to determine the best feeding practices in this population [45].

The rate of vertical transmission of CMV in twin pregnancies is higher, approximately 58.7% (95% CI 43.3 to 72.3%), and the discordance of positivity between twins is approximately 50%. The concordance between infected twins is higher in monozygotic pregnancies than in dizygotic pregnancies (84.6% vs. 37.5%), suggesting possible genetic susceptibility to CMV [41].

### 4.2. Pathophysiology

Human CMV is a host-restricted, endemic, ubiquitous member of the herpes virus family [11]. It has a large double-stranded DNA genome of 236 kb that encodes at least 167 gene products, over 40 of which are involved in the host immune response. After primary infection, CMV becomes established for life and has multiple mechanisms for attacking the immune system [11,61].

Congenital CMV infection can damage the fetus directly or indirectly through placental dysfunction resulting from infection or immune-mediated destruction, leading to miscarriage, preterm birth, or fetal growth restriction (FGR) [11]. Njue et al. [11] published a review of the possible mechanisms by which CMV could cause indirect damage to the fetus, leading to FGR, miscarriage, and preterm birth. The study also noted that the pathophysiology of CMV is not fully understood, and the studies reviewed that attempted to understand this mechanism were developed in vitro.

In vitro, CMV was able to invade several placental cells. The trophoblastic progenitor stem cell (precursor of the syncytium and cytotrophoblast), which reduces the number of mature cells, the extravillous trophoblast (floating cytotrophoblast), which is responsible for invading the uterine vascular wall and for the process of remodeling the circulation during pregnancy, its destruction would lead to deleterious effects on the pregnancy due to the reduction of maternal blood circulation in the placenta and consequent reduced fetal access to nutrients, leading to fetal growth restriction or even miscarriage [11]. The exact consequences of placental cell invasion are unknown and vary with gestational age [11]. It is thought that infection shortly after implantation may disrupt this process and lead to miscarriage. Destruction of the cells responsible for vascular invasion would lead to fetal growth restriction, and destruction of anchoring cells would lead to preterm birth [11].

CMV also interferes with key autoregulatory pathways in the cytotrophoblast, which would alter trophoblast migration, and has immunomodulatory properties that alter the host immune response. These alterations could lead to miscarriage, fetal growth restriction, and preterm labor. In vitro, CMV increases tumor necrosis factor-alpha levels, leading to accelerated trophoblast apoptosis, which is another way to explain growth restriction in fetuses unaffected by CMV whose mothers were diagnosed with the disease [11].

Susceptibility to fetal infection increases with gestational age, probably due to the process of cytotrophoblast differentiation. The virus eventually crosses the placenta, the first fetal organ to be infected, and replicates in the tubular epithelium of the fetal kidney, with tropism for reticuloendothelial cells and the central nervous system (CNS) [13]. The probable sequence of events (taking 7 to 8 weeks) leading to fetal infection is maternal viremia, placental infection, and fetal dissemination via the hematogenous route [17].

### 4.3. Ultrasonographic and Magnetic Resonance Imaging Findings

**Figure 2:**
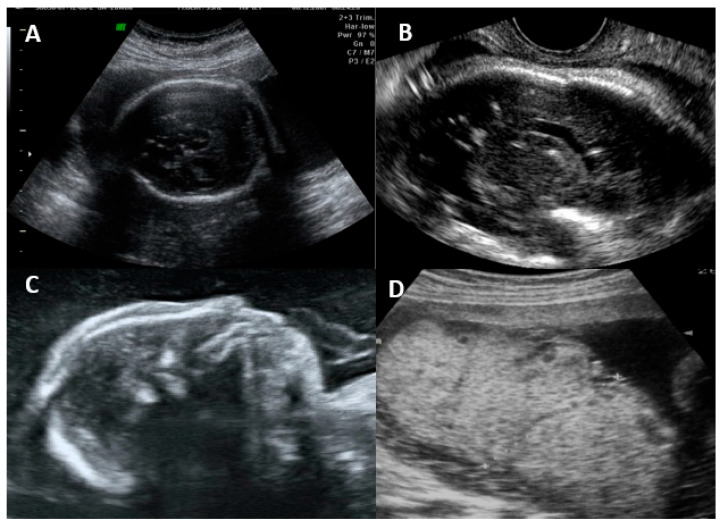
Ultrasonographic findings in a fetus with intrauterine cytomegalovirus infection. (**A**) Transabdominal ultrasound showing periventricular calcifications. (**B**) Transvaginal ultrasound showing periventricular calcifications. (**C**) Microcephaly. (**D**) Placentomegaly.

The fetal disease is usually progressive, and the initial symptoms on ultrasonography are usually due to systemic infection (FGR, abnormal amniotic fluid volume, ascites, pleural effusion, skin edema, hydrops, placentomegaly, hyperechogenic bowel, splenomegaly, and hepatic calcifications) [16,55]. CNS findings usually occur after weeks, and severe brain involvement is usually a predictor of poor prognosis, with microcephaly being the only finding that actually predicts an unfavorable outcome in up to 95% of cases [16,25,55]. The most common findings and their classification are summarized in Table 2. Figure 2 shows the ultrasonographic findings regarding intrauterine CMV infection.

Vasculitis is a nonspecific finding described as a candlestick pattern of punctate echogenicity within the brain parenchyma underlying the lateral ventricular rim, along with strands within the ventricle [56]. Hyperechogenic intestine is the most common extracerebral finding, but it is nonspecific and can occur in normal fetuses, those with chromosomal abnormalities, cystic fibrosis, and other infections. Most cases resolve spontaneously; however, fetuses with this finding, even if isolated, should be evaluated and followed [49].

**Figure 3:**
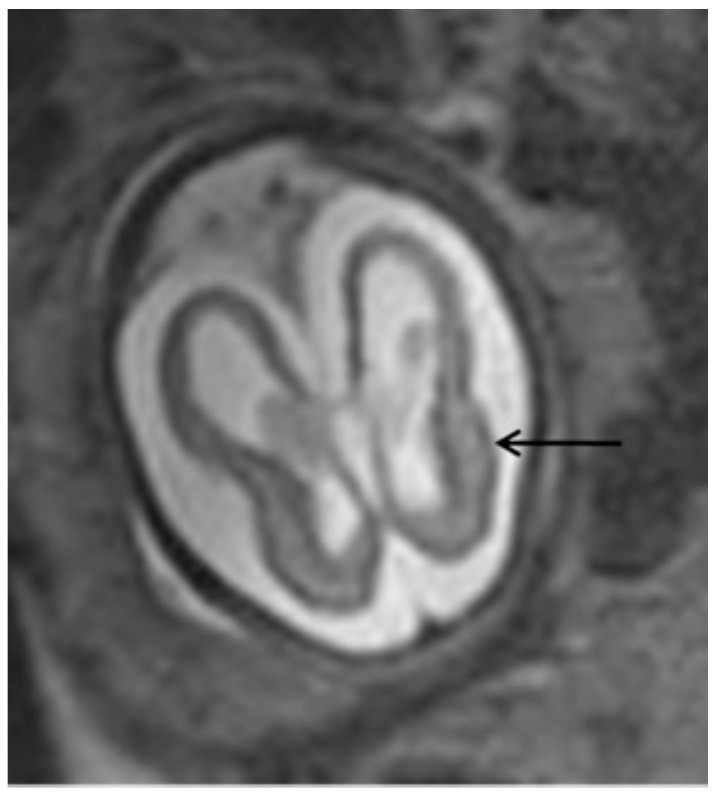
T2-weighted MRI in axial view of fetal skull showing mild ventriculomegaly and cortical atrophy (arrow).

The most common ultrasonographic findings of intrauterine CMV infection are ventriculomegaly, periventricular changes, temporal cysts, and brain parenchymal lesions [25]. Magnetic resonance imaging (MRI) has been shown to complement ultrasonographic imaging in prenatal CMV assessment [25,26]. For example, Buca et al. [26] found that in 6% of ultrasounds in which no CNS abnormalities were seen, the MRIs were positive, but only in cases infected in the first trimester of pregnancy. The abnormalities seen on MRI are the same as those seen on ultrasound, but with a different incidence, with the most common abnormalities seen on MRI being temporal cysts and lesions in the brain parenchyma [25]. (Figure 3) It is known that the additional findings on MRI are due to the lack of studies using multiplanar neurosonography performed by an experienced professional, because when this is method is used, the results of both types of imaging are similar [26,27]. Intrauterine CMV infection is an indication for multiplanar (transvaginal) neurosonography [27]. The main changes seen on neurosonography are abnormal patterns of periventricular echogenicity (suggesting periventriculitis), ventriculomegaly, and echogenic focus in the brain parenchyma [27].

**Figure 4:**
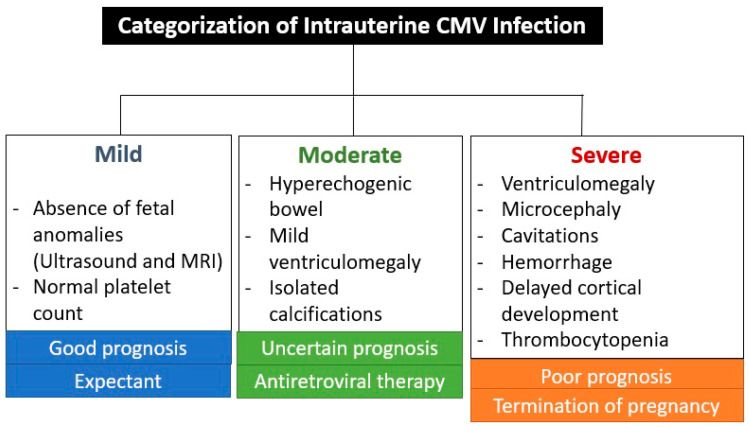
Categorization of intrauterine cytomegalovirus infection according to Khalil et al. [31].

The absence of CNS ultrasound and MRI abnormalities during prenatal care is an important prognostic factor, as a very small percentage of newborns are symptomatic (1.5%) or have abnormal neurodevelopment (3.1%) or hearing loss (up to 11.4%) [26]. These abnormalities are associated with seroconversion in the first trimester of pregnancy, except for hearing impairment, which also occurred with seroconversion in the second trimester in 7% and in the third trimester in 0% [26]. CNS abnormalities seen on ultrasound and MRI are not specific to CMV infection, but are indicative of intrauterine infection [26,27]. The virus reaches the brain via the hematogenous route, entering the cerebrospinal fluid and causing inflammation of the choroid plexus and meninges [27]. This is the reason why the ventricular and periventricular lesions appear before the brain parenchyma lesions [27]. Figure 4 shows the categorization of intrauterine CMV infection according to Khalil et al. [31].

Termination of pregnancy in cases of intrauterine CMV infection is accepted by professionals in 90% of cases with severe changes. If there are no changes on ultrasound, 94% contraindicate abortion, and if there are mild changes, 78% contraindicate abortion [44].

## 5. Diagnosis

The matter is the same with regard to pregnant women; there are no guidelines for universal testing for congenital CMV infection in newborns [2]. Diagnosis in the fetus is made from amniotic fluid by positive culture or by polymerase chain reaction (PCR) [2,12]. The sample should be collected after 21 weeks’ gestation and between 6 and 8 weeks after maternal infection to reduce the risk of a false negative result [3,12]. Amniocentesis shows sensitivity of approximately 86% and specificity of 100%, with a positive predictive value of 100% and a negative predictive value of 95% [12]. Rarely, a false positive sample can occur due to contamination with maternal blood [14].

Detection of CMV in newborns is performed by viral detection in body fluids (urine, saliva, and blood) by PCR, culture, or antigen testing (pp65 antigen) up to 3 weeks of life [2]. After this period, it is difficult to differentiate congenital from acquired postnatal infection [2]. Detection of IgM for CMV in newborns during the same period may also be considered [14]. Urine and saliva specimens are more sensitive than blood for the detection of CMV [47,54]. The presence of the virus in urine and saliva lasts the same amount of time, but the number of copies is 10 times higher in saliva than in urine [61].

## 6. Prognosis

Chatzakis et al. [13] published a meta-analysis on the fetal outcomes of intrauterine and postnatal maternal primary infection, finding that although vertical CMV transmission increased with gestational age by 5.5%, 21%, 36.8%, 40.3%, and 66.2% for the preconception period (up to 12 weeks before the last menstrual period), the periconception period (4 weeks before to 3–6 weeks after last menstrual period), the first trimester, the second trimester (14 to 26 weeks), and the third trimester, respectively, fetal abnormalities were limited to infection acquired periconceptionally and in the first trimester. The fetal insult rates (fetal insult was considered to be any CNS malformation on ultrasound or that led to termination of pregnancy or findings of neurological symptoms at birth) in cases of vertical transmission were 28.8%, 19.3%, 0.9%, and 0.4%, respectively, for the periconception period, first trimester, second trimester and third trimester [13].

The outcomes of sensorineural hearing loss and/or delayed neuropsychomotor development found by Chatzakis et al. [13] also varied with the gestational age of infection, being 22.8%, 0.1%, and 0% for the first, second, and third trimesters, respectively. Symptoms at birth could also be correlated with the period of vertical transmission and were found in the following proportion in newborns: 1.3%, 9.1%, 0.3%, and 0.4%, respectively, for the periconception period, first trimester, second trimester and third trimester.

Pre- and periconceptional infection is explained by the fact that maternal viremia peaks about 7 weeks after primary infection and lasts up to 12 weeks [13]. Brain malformations are also associated with the gestational age of infection: microcephaly < 18 weeks, polymicrogyria between 18 and 24 weeks, and normal rotation with diffuse white matter heterogeneity correspond to infection in the third trimester [13].

The platelet count at cordocentesis has been shown to be an important prognostic factor, where fetuses with a platelet count <50,000/mm^3^ have an 80% risk of poor prognosis (termination of pregnancy, miscarriage, fetal death, or CNS sequelae) [16,24]. In addition to platelets, high fetal viremia and high fetal ß2-microglobulin counts are associated with more severe disease [24].

Newborns with a negative amniocentesis have a lower risk of developing symptomatic infection (4.3% vs. 25%) and hearing loss (2.2% vs. 17.4%). In the study by Dinsmoor et al. [12], none of the children with negative amniocentesis developed neurological sequelae, compared with 14.1% of positive children. The presence of virus in amniotic fluid is also associated with low birthweight and preterm birth [12]. In a meta-analysis published in 2023, Chatzakis et al. [23] showed that when amniocentesis was negative for CMV, 0% of neonates had severe neonatal symptoms, severe sensorineural hearing loss, and/or delayed neurological development, or termination of pregnancy due to CNS or systemic imaging findings associated with the presence of CMV. The absence of sequelae was maintained even in neonates who had a positive urine sample for CMV (this occurs in up to 8% of cases with negative amniocentesis PCR).

The risk of an infected fetus being symptomatic at birth can be estimated from prenatal imaging results and laboratory tests [16]. Table 3 shows the main findings and factors that may predict a poor prognosis.

**Figure 5:**
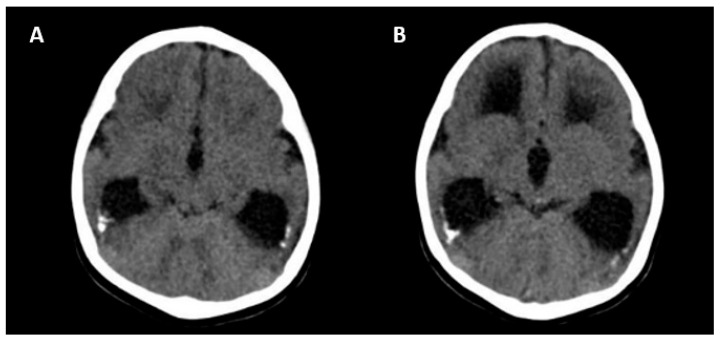
Computed tomography axial view of a newborn skull with intrauterine cytomegalovirus infection showing periventricular calcifications (**A**) and moderate ventriculomegaly (**B**).

The absence of CNS abnormalities on ultrasound and MRI is associated with a good prognosis [25]. Unfavorable outcomes include the following: neurological symptoms (tetraplegia/cerebral palsy, lethargy and/or hypotonia, chorioretinitis, sensorineural hearing loss, microcephaly and delayed neuropsychomotor development), abnormal CNS imaging findings at birth, hematological alterations (thrombocytopenia/liver enzymes), and termination of pregnancy due to fetal malformations [25]. Figure 5 shows the CNS abnormalities using computed tomography in a newborn with intrauterine CMV infection.

## 7. Symptomatic Newborns

### 7.1. After Delivery

Ten to fifty percent of newborns delivered to mothers with primary CMV infection are symptomatic, compared with 1% of newborns delivered to mothers with non-primary infection [1,2,6,10]. Clinical findings of intrauterine infection include FGR (50%), jaundice (67%), hepatosplenomegaly (60%), generalized petechiae (76%), purpura, thrombocytopenia (77%), hydrops, pneumonitis, microcephaly (53%), abnormal brain imaging (calcifications, periventricular hyperinflammation, ventriculomegaly, subependymal cysts, and striated lenticular vasculopathy), seizures (7%), chorioretinitis, hearing loss, bone abnormalities, abnormal dentition, anemia, hypotonia/lethargy (27%), arterial hypertension, and CMV isolated in the cerebrospinal fluid [2,6,10,12].

McCarthy et al. [14] classified neonatal symptoms as severe or moderate. Any change in the CNS at birth, such as microcephaly, CNS imaging showing calcifications or white matter abnormalities, sensorineural hearing loss (any degree, unilateral or bilateral), or chorioretinitis, was considered severe; all others were considered moderate. According to Maltezou et al. [10] in a meta-analysis published in 2020, the difference in severity between primary and non-primary maternal CMV infection was not confirmed in studies conducted with universal screening for newborns, either for symptoms at birth or for the development of sequelae (unilateral or bilateral sensorineural hearing loss or another neurological outcome). This finding may suggest that universal screening for newborns can be a perspective [10].

### 7.2. Long-Term Sequelae

Long-term sequelae can occur in symptomatic (40–60%) or asymptomatic (+/−13.5%) congenital CMV infections, though symptomatic infections are more frequent and severe [2,13,48,53]. Twenty-five percent of asymptomatic newborns of mothers with primary infection are at risk of developing sequelae in the following 24 months, compared to 8% of mothers with non-primary infection [6,13]. The most common sequelae are sensorineural hearing loss, vision loss, mental retardation, seizure disorders, cerebral palsy, visual abnormalities (chorioretinitis, optic atrophy, cortical visual impairment, and strabismus), or delayed neuropsychomotor development [2,14].

Sensorineural hearing loss following symptomatic or asymptomatic infection is usually progressive, unilateral or bilateral, and possibly absent at birth but then manifesting later in childhood [2,48,51]. Bilateral hearing loss is more common when the newborn is symptomatic [53]. Approximately 21% of hearing loss at birth and 25% by 4 years of age are attributed to congenital CMV infection [2]. Between 6% and 23% of asymptomatic newborns may have lifelong sensorineural hearing loss [6,46]. It is believed that approximately 5% of children who develop microcephaly or have delayed neuropsychomotor development have undiagnosed CMV [6,46].

Although sensorineural hearing loss is the most common type, vestibular dysfunction can occur in symptomatic and asymptomatic newborns with or without hearing loss [43]. Newborns, infants, and children with congenital CMV infection have poorer outcomes in psychological development, sequential and simultaneous processing, phonoaudiological working memory, motor performance, social communication, and attention control compared to age-matched controls. It is concluded that congenital CMV is associated with cognitive decline in young populations [40].

In addition to hearing loss and developmental delay, congenital CMV infection can cause ophthalmic disorders (chorioretinitis, cataracts, strabismus, optic nerve atrophy, and retinal hemorrhage), the main one being chorioretinitis [60]. For this reason, these children should have a fundus examination at birth and follow-up with a specialist [60,62].

## 8. Primary Prevention

According to McCarthy et al. [14], there is insufficient evidence regarding actions capable of eliminating the risk of vertical CMV transmission, whether medication or behavioral measures (hand hygiene, wearing gloves to change diapers, and washing dirty clothes). One analytical model indicated that personal hygiene is highly effective in preventing unfavorable outcomes in congenital CMV infection, showing a 50% reduction in the rate of infection in seronegative populations [9]. However, some studies have suggested that maternal adherence may be a limiting factor [29]. The main difficulty is not having intimate contact (kissing lips; sleeping together; or sharing cutlery, food and drinks) with younger children [28,29,32,53].

Pregnant women are more motivated to accept behavioral changes to protect the health and development of the fetus compared to non-pregnant women. In addition, better hygiene habits can prevent other diseases [33]. In a randomized study in which pregnant women susceptible to CMV watched a video explaining the risks of the infection, what it was, and how to avoid it, there was no difference compared to the control group. However, the authors attributed the result to the small number of participants [28]. Although behavioral measures are important tools in protecting against CMV, some studies have shown that only 22% of women are informed about CMV during pregnancy and its possible consequences, and only 50% of obstetricians advise pregnant women about it [10,14,29].

There is currently no CMV vaccine available, although many studies are underway and it is considered a priority [9,16,17,18,19,20,21]. The development of a vaccine against CMV is complicated due to the various properties that make the development of protective immunity and safety a challenge. Among these properties is the ability of the virus to establish latent infection after resolution of the primary infection by circumventing the host’s immune system. Studies have shown that a CMV vaccine may not be able to completely prevent infection against circulating strains, but it could prevent recurrent infection in most women, consequently providing protection against vertical transmission. In theory, the maternal response to the vaccine would produce IgG that crosses the placenta and neutralizes CMV, preventing it from invading fetal cells [14].

To date, most protocols do not suggest universal screening and/or treatment of CMV, so interventions to reduce the risk of vertical transmission are limited to behavioral measures (washing hands, avoiding contact with urine and saliva of young children, etc.) [9].

## 9. Treatment

Antivirals against CMV, including ganciclovir, valganciclovir, cidofovir, and foscarnet, have been shown to be effective in non-pregnant adults. Pharmacologically, these drugs inhibit CMV replication at the cellular level by various processes [14]. However, these drugs are not licensed for use during pregnancy.

Ganciclovir is not well absorbed in the gastrointestinal tract (only 8%), while valganciclovir is well absorbed [15]. Ganciclovir cannot be used during pregnancy due to the risk of toxicity to fetal germ cells [19]. Valacyclovir is a prodrug of acyclovir and is transformed into acyclovir in the first hepatic passage. Valacyclovir has been widely used instead of acyclovir in the treatment of herpes virus infection because it is more effective [18]. Following oral administration of valacyclovir, <1% is excreted unchanged, and >85% is excreted as acyclovir through glomerular filtration and active tubular secretion [18,20]. Valacyclovir is classified as category B in pregnancy [20]. Valacyclovir is a precursor to acyclovir, a DNA polymerase inhibitor. Acyclovir is subsequently converted to acyclovir triphosphate, which itself exerts antiviral activity via DNA strand breaks and disruption of viral DNA synthesis [22].

Valacyclovir has been the most widely used, widely studied, and promising medication for preventing congenital CMV infection after primary maternal infection in early pregnancy. It can also be offered to mothers whose fetuses show ultrasonographic alterations compatible with intrauterine CMV infection and proven via maternal serology or PCR in the amniotic fluid [16,18]. However, more studies are needed to support this type of use of valacyclovir [20]. Some authors have discussed the administration of this medication for primary infection acquired in the second trimester, as it may reduce the risk of symptoms at birth and long-term sequelae [16,18,19]. Egloff et al. [17] published a retrospective study in 2021 comparing vertical transmission between treated and untreated patients with primary infection acquired in the second trimester, and the rate of vertical transmission was 25% in treated patients vs. 58% in untreated patients.

Human hyperimmune globulin, another option suggested in some recent studies, is extracted from human plasma from selected donors and has antiviral and immunomodulatory properties [14]. Ganciclovir can penetrate various body compartments, including transplacental passage and penetration into the cerebrospinal fluid of newborns [15].

Valacyclovir is contraindicated in patients who are unable to swallow capsules; with hyperemesis gravidarum (severe vomiting after starting the medication); with pre-existing liver disease, renal dysfunction, or bone marrow suppression; on immunosuppressive therapy; or with known hypersensitivity to acyclovir [17]. Most of the studies analyzed suggest that the rate of serious adverse events is low, about 2.1 to 3.17%, the most common being acute renal failure, which resolved spontaneously after discontinuation of the medication [19,20,21,22]. Other side effects reported were back pain, dyspepsia, nausea, dizziness, and macrocytosis [19].

### 9.1. Intrauterine (Primary Prevention)

Until about a decade ago, there were no protocols that suggested intrauterine treatment of congenital CMV infection, as stated by McCarthy et al. [14] This situation has gradually changed due to strong evidence that treatment with valacyclovir improves the prognosis of the fetus and newborn. Leruez-Ville et al. [16] published a study which, although not randomized, demonstrated the antiviral efficacy of valacyclovir in infected fetuses. When high-dose valacyclovir (8 g/day = 16 tablets/day) was given to the mother until delivery or for 24 weeks (whichever came first), there was a reduction of symptoms in newborns from 43% (meta-analysis obtained from the literature) to 82%. In addition to the drop in the percentage of asymptomatic births, follow-up with cordocentesis showed an increase in platelet count and a decrease in fetal viral load [16]. Children who were born asymptomatic were followed up for 12 months, and those who were born symptomatic were treated with valganciclovir and none had sensorineural hearing loss during the same period [16]. It is important to note that this study excluded asymptomatic fetuses and those with brain alterations considered severe.

In 2020, Shahar-Nissan et al. [17] published a double-blind randomized study using valacyclovir (8 g/day) to prevent congenital CMV infection acquired periconceptionally or in the first trimester of pregnancy. Although the number of participants was low (90 in total), the results were encouraging in terms of the value of valacyclovir. In the group taking valacyclovir who acquired the infection in the first trimester, PCR was positive in the amniotic fluid in 11% of cases (2/19) versus 48% in the placebo group (11/23), with no difference between the groups when considering periconceptional infection, possibly due to starting treatment further away from the contamination period in the latter group. Pregnant women were treated from recruitment until the date of amniocentesis (21 weeks) or for at least 7 weeks after the estimated date of primary infection [17].

In 2023, Amir et al. [21] published a revised protocol for initiating valacyclovir therapy. They started at a maximum of 8–9 weeks from the presumed time of infection in the case of periconceptional infection and at a maximum of 18 weeks in infections acquired in the first trimester of pregnancy. With this revised protocol, vertical transmission was also lower in pregnant women with periconceptional infection (valacyclovir 0/59 vs. 3/24 for those who received a placebo) when PCR in amniotic fluid was considered.

After Shahar-Nissan’s study, several other studies confirmed the benefits of valacyclovir in preventing congenital CMV infection [3,18,19,20,21,22]. In an observational study carried out by Zammarchi et al. [3] in Italy with 447 pregnant women with primary CMV infection acquired from the periconceptional period up to 24 weeks of gestation, 205 received treatment and 242 did not. The result was a statistically significant reduction in the amniocentesis positivity rate (treated 14.7% vs. untreated 27.6%), the rate of symptomatic newborns at birth (treated 1.6% vs. untreated 8.9%), and the number of terminations of pregnancy (treated 3.4% vs. untreated 9.8%). However, there was no significant reduction in the prevalence of CMV DNA detection in the urine (treated 22.2% vs. untreated 25.3%).

The adverse effects of valacyclovir include headache, gastrointestinal symptoms (vomiting and abdominal pain), kidney toxicity, fatigue and dizziness, and skin rash [3,17]. Valacyclovir is well tolerated, even at high doses, and there has been no association with fetal malformations in pregnant women previously exposed to the medication [16,17,19]. Table 4 shows the interpretation of maternal serology results up to 14 weeks to determine the period of infection or serological status.

Studies differ on how long valacyclovir should be administered. Some authors, such as Shahar-Nissan et al. [17], opted for discontinuation after amniocentesis, while others, such as Egloff et al. [19], suggest that valacyclovir treatment should be continued until the end of pregnancy to prevent late transmission and its consequences (usually sensorineural hearing loss in up to 4.3% of cases). The latter option has not been supported by prospective randomized trials.

CMV-specific hyperimmune globulin (anti-CMV IgG antibody) was studied in 2005 in a non-randomized trial to prevent vertical transmission [32]. In 2014, Revello et al. [32] published the first randomized trial of this drug and concluded that there was no benefit from its use, with an infection rate of 30% in the group using the globulin and 44% in the control group. In this trial, there was an increase in preterm birth and low birthweight rates in the group using the globulin.

In 2021, a randomized phase 3 trial was published on the use of immunoglobulin, and although it reached the required sample size, it did not show a significant reduction in vesicle transmission [34]. In 2020, El-Qushayri et al. [35] published a meta-analysis in which hyperimmune globulin was effective in preventing congenital CMV infection in cases of maternal primo-infection, but was not effective in treating CMV. The most common adverse effects found were FGR, preterm delivery, and termination of pregnancy. Another fact highlighted by the authors is that the dose of globulin varied in most of the included studies, namely between 100, 150, and 200 U/kg per month [35].

### 9.2. Newborn (Tertiary Prevention)

Treatment of congenital CMV infection may be considered in newborns who are symptomatic at birth, who have CNS involvement (including hearing loss), and who have severe disease, such as hepatitis, pneumonia, or thrombocytopenia [2]. The drug of choice is ganciclovir, which can be started intravenously in the first month of life at 6 mg/kg/day for 42 days, with the need for a central catheter [2]. Although well tolerated and considered safe, ganciclovir can cause neutropenia (60% of cases), which is easily reversed with human granulocyte colony-stimulating factor. Dose adjustments should be made for newborns with varying degrees of renal insufficiency [2].

Ganciclovir has shown very good results, both in terms of better neuropsychomotor development and less hearing loss in the short-term and especially in the long term, in treated newborns compared to untreated ones. It is important to note that there is no improvement in already-established lesions [2]. This fact suggests that the reduction in CMV viral load with treatment during the period when the newborn brain is most susceptible to damage is the most likely cause of the better outcomes in treated newborns [15].

**Figure 6:**
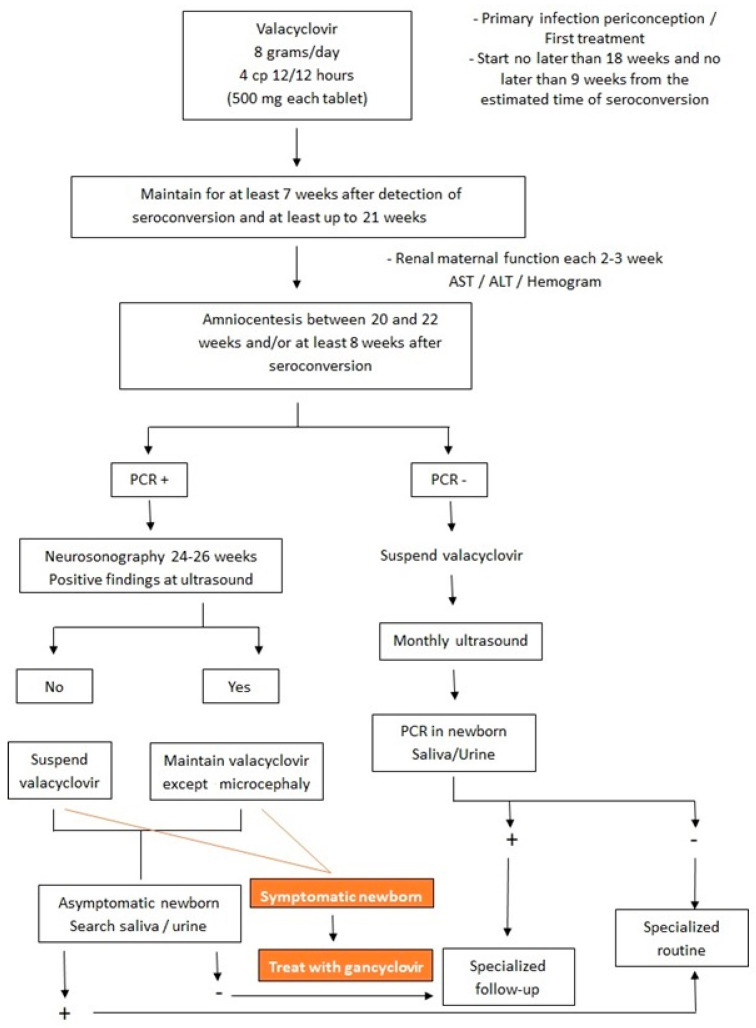
Flowchart of treatment in both fetus and newborn with intrauterine cytomegalovirus infection.

Despite the improved long-term prognosis with ganciclovir treatment, the child will shed the virus in saliva and urine after treatment is completed [2]. Some studies have suggested valganciclovir as an alternative treatment for congenital CMV, with the advantage that it is taken orally. The dosage would be 16 mg/kg/dose every 12 h for 42 days. However, more studies are needed [2]. Figure 6 shows the flowchart of treatment in both fetus and newborn with intrauterine CMV infection.

## 10. Conclusions

Primary or non-primary maternal infection is poorly diagnosed because in most countries, CMV testing during antenatal care and in the newborns is not indicated and/or not available. Non-primary maternal infection, despite rare intrauterine transmission, is a significant cause of long-term sequelae when considered in the population as a whole, due to the high prevalence of CMV in the world population [10,56]. Because most women are immunocompetent, primary CMV infection causes few or no symptoms, so the exact time of infection is estimated only via serologic testing [13]. Ultrasound and MRI findings are nonspecific and cannot predict outcomes except in cases of microcephaly.

Given the new evidence that valacyclovir is a safe, well-tolerated drug and reduces vertical transmission, protocols for pregnant women that do not recommend testing for CMV during pregnancy need to be revised [56]. Previously, universal testing of pregnant women was not indicated because there was no therapeutic option. Currently, high-quality evidence supports the use of valacyclovir in women with periconceptional or first-trimester CMV infection [36]. Treatment with human hyperimmune globulin, on the other hand, has not been supported in the literature [36,37]. Most studies are observational, and the aforementioned randomized phase 3 trial did not reach the required number of participants and was discontinued after 8 years [34,36].

Implementing routine screening for CMV as early as possible would be an important tool to understand the disease in future studies, start treatment at an appropriate gestational age, refer the pregnant woman to a specialized center, determine the gestational age of seroconversion, and allow for appropriate referral of the newborn, which is not always possible [58].

The lack of effective primary prevention, either due to behavioral measures or the lack of a vaccine, makes valacyclovir, if started as soon as possible in the case of primary maternal CMV infection diagnosed in the first trimester of pregnancy, the best option for reducing the risk of health problems caused by congenital CMV, either at birth or in the long-term follow-up. Another point to be considered is that 50% of instances of neurological sequelae in cases of congenital CMV infection come from non-primary infections [22]. In the event of reactivation, asymptomatic newborns should be tested, and symptomatic newborns should be treated if transmission is confirmed by available tests. If primary and secondary prevention fail, it is still possible to treat symptomatic newborns with ganciclovir, with strong evidence of improvement in both short- and long-term prognoses [2,15,29].

Despite the fact that congenital CMV infection has serious consequences and is very common in absolute numbers, pregnant women generally have little or no knowledge of this disease, and the doctors and nurses who care for these patients are not in the habit of advising them on primary prevention. All pregnant women and women who intend to become pregnant need to be informed about this disease and about ways to improve hygiene to reduce transmission [38,52].

In summary, the main explanation for not implementing CMV serologic testing in pregnant women was the lack of an effective, safe, and available treatment. As new studies have showed promising treatment with high doses of valacyclovir in cases of seroconversion in the periconceptional and first trimester of pregnancy periods, we believe that the new guidelines should be amended to include the screening for CMV in the periconceptional and first trimester of pregnancy periods.

## Figures and Tables

**Table 1:** Criteria for classifying maternal infection in the case of suspected or confirmed congenital infection.

Type of Infection	Definition
Confirmed primary infection	IgG and IgM− previously, showing serum conversion during pregnancy *
Presumed primary infection	CMV IgG+, with low avidity ** and IgM+, in the first trimester or CMV IgG and IgM+, with undetermined IgG avidity, with detection of CMV-DNA in at least 1 body fluid (blood, urine, or saliva) during pregnancy
False positive	IgM+ and IgG− in paired tests with a difference of at least 2 weeks *
Confirmed non-primary infection	CMV IgG+ before pregnancy or CMV IgG+ and IgM− in the 1st trimester
Presumed non-primary infection	CMV IgM− and 1- IgG+ before 12 weeks with unknown IgM or 2- Four times increase in IgG titers in paired tests
Congenital CMV infection	Detection of CMV (culture) or CMV-DNA via PCR in the newborn’s saliva, urine, or blood obtained up to 3 weeks of age or in the amniotic fluid [2]

CMV: cytomegalovirus; PCR: polymerase chain reaction. * IgM immunoglobulin can cross-react with other viruses, such as Epstein–Barr, for example, so for seroconversion to be confirmed, IgG positivity is required in paired tests [9]. ** IgG avidity test < 15% suggests infection at less than 6 weeks and <35% at less than 12 weeks [17]. Avidity > 65% considers >12 weeks of infection and between 40% and 65% is considered indeterminate [18]. These values may vary according to methodology and laboratory.

**Table 2:** Ultrasonographic findings of intrauterine cytomegalovirus infection, based on the criteria of Leruez-Ville et al. [16].

Extra-CNS	
	FGR Abnormal amniotic fluid volume Ascites and/or pleural effusion Skin edema Hydrops Placentomegaly > 40 mm Hyperechogenic intestines Hepatomegaly > 40 mm (right lobe) Splenomegaly > 40 mm (largest diameter in the second trimester) Hepatic calcifications Cardiomegaly
**Moderate CNS malformations**	
	Moderate ventriculomegaly < 15 mm Isolated cerebral calcification Isolated interventricular adhesion Vasculopathy/hyperechogenicity of lenticulostriate vessels
**Severe CNS malformations**	
	Ventriculomegaly > 15 mm Periventricular hyperechogenicity Hydrocephalus Microcephaly < 3 SD Mega cisterna magna > 10 mm Hypoplasia of vernix or cerebellum Porencephaly Lissencephaly Periventricular cysts Corpus callosum abnormality

CNS: central nervous system; FGR: fetal growth restriction; SD: standard deviation.

**Table 3:** Predictors of poor neonatal prognosis in intrauterine cytomegalovirus infection.

Criteria for Poor Intrauterine Prognosis	
**Cordocentesis**	
	Viral load > 30,000 copies/mL Platelets < 50,000 mm^3^ Increased ß2-microglubulin High levels of specific IgM
**Ultrasound or MRI**	
	Microcephaly
**Time of maternal infection**	
	Periconceptional-4 weeks before the last menstrual period up to 3 weeks of gestation * First trimester
**Amniocentesis**	
	Positive PCR for CMV with high viral replication

CMV: cytomegalovirus; MRI: magnetic resonance imaging: PCR: polymerase chain reaction. * The definition of the periconceptional period varies in the literature from 4 weeks before the last menstrual period to 3 to 6 weeks after.

**Table 4:** Interpretation of maternal serology results for cytomegalovirus up to 14 weeks to determine the period of infection or serological status.

Period of Primary Infection or Other Serological Status		CMV-Specific Antibodies	
	**IgG**	**IgM**	**IgG Avidity**
>12 weeks	+	+	High
Periconceptional infection	+	+	Intermediate
Infection in the first trimester of pregnancy *	+	+	Low
	−	+	x
No prior contact	−	−	x
False positive test **	−	+	x

CMV: cytomegalovirys; * IgG positive after paired tests with 15 days between them; ** IgG remains negative in paired tests with a difference of at least 15 days.

## Data Availability

The data presented in this study are available on request from the corresponding author.
